# The mammalian ULK1 complex and autophagy initiation

**DOI:** 10.1042/EBC20170021

**Published:** 2017-12-12

**Authors:** Maria Zachari, Ian G. Ganley

**Affiliations:** MRC Protein Phosphorylation and Ubiquitylation Unit, School of Life Sciences, University of Dundee, Dundee DD1 5EH, U.K.

**Keywords:** autophagy, kinase, ULK1

## Abstract

Autophagy is a vital lysosomal degradation pathway that serves as a quality control mechanism. It rids the cell of damaged, toxic or excess cellular components, which if left to persist could be detrimental to the cell. It also serves as a recycling pathway to maintain protein synthesis under starvation conditions. A key initial event in autophagy is formation of the autophagosome, a unique double-membrane organelle that engulfs the cytosolic cargo destined for degradation. This step is mediated by the serine/threonine protein kinase ULK1 (unc-51-like kinase 1), which functions in a complex with at least three protein partners: FIP200 (focal adhesion kinase family interacting protein of 200 kDa), ATG (autophagy-related protein) 13 (ATG13), and ATG101. In this artcile, we focus on the regulation of the ULK1 complex during autophagy initiation. The complex pattern of upstream pathways that converge on ULK1 suggests that this complex acts as a node, converting multiple signals into autophagosome formation. Here, we review our current understanding of this regulation and in turn discuss what happens downstream, once the ULK1 complex becomes activated.

## Introduction

**Figure 1:**
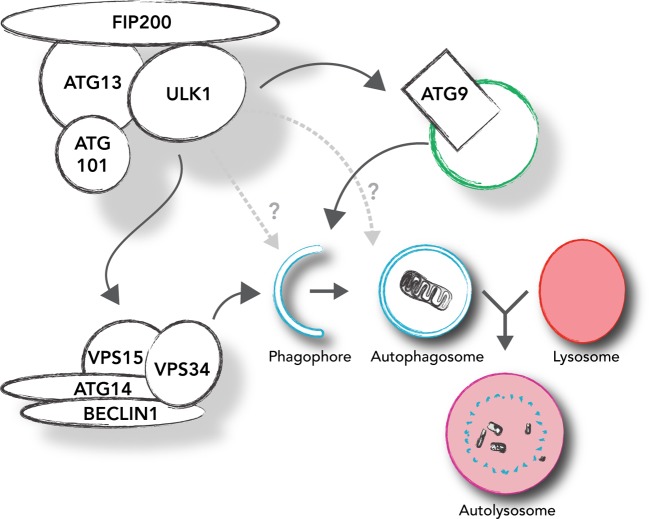
Role of ULK1 (unc-51-like kinase 1) in autophagy induction Schematic highlighting our current understanding of autophagy initiation. The ULK1 complex drives formation of the phagophore, the initial autophagosomal precursor membrane structure, through direct activation of the VPS34 (vacuolar protein sorting 34) complex and by mediating trafficking of ATG (autophagy-related protein) 9 (ATG9). As discussed in the text, it is also likely that ULK1 has additional roles in autophagosome maturation. Once cargo has been engulfed, the autophagosome fuses with the lysosome to form the digestive autolysosome where cargo is degraded and recycled.

Macroautophagy (hereafter autophagy) is a major catabolic pathway involving the generation of a double-membrane, vesicle-like autophagosome in which cytoplasmic components, be they protein complexes or whole organelles, are sequestered and delivered to lysosomes for degradation (see Figure 1). Autophagy is an evolutionarily conserved homoeostatic process that serves as a quality control mechanism as well as a recycling pathway. It can be rapidly activated under conditions of stress to prevent the accumulation of detrimental cellular components or to allow bulk recycling of cellular constituents under starvation conditions. As this is a lysosomal pathway, autophagy can mediate not just protein degradation but also that of lipid, carbohydrate and nucleic acids. Dysregulation of the pathway has been linked with numerous human disorders including neurodegenerative diseases such as Parkinson’s and Alzheimer’s, as well as cancer [1,2].

Initiation and formation of an autophagosome is a complicated process, orchestrated by three main protein complexes. The very first autophagy-specific complex that comes into play is the ULK1 (unc-51-like kinase 1) complex, consisting of ULK1 itself, ATG (autophagy-related protein) 13 (ATG13), FIP200 (focal adhesion kinase family interacting protein of 200 kDa), and ATG101. Upon autophagy induction, the ULK1 complex translocates to autophagy initiation sites and regulates the recruitment of a second kinase complex, the VPS (vacuolar protein sorting) 34 (VPS34) complex. The VPS34 complex consists of the class III phosphatidylinositol 3-kinase VPS34, as well as BECLIN-1, VPS15 and ATG14L (ATG14-like). This complex is responsible for the production of the phospholipid phosphatidylinositol 3-phosphate (PI3P) at the site of forming autophagosome, termed as the phagophore, which acts as a signalling molecule for the recruitment of PI3P-binding proteins such as WIPI2B (WD repeat domain phosphoinositide-interacting protein 2) and DFCP1 (double FYVE containing protein 1). These and other proteins then lead to formation and expansion of the phagophore, which eventually seals to form the complete autophagosome. The molecular details of this step are still far from being solved, but they involve the recruitment of the third protein complex, consisting of the ATG16L1–ATG5–ATG12 conjugation machinery. This complex is believed to act in an analogous fashion to ubiquitin E3 ligases by catalysing the conjugation of the ubiquitin-like ATG8 family (LC3A, B and C (microtubule-associated protein 1 light chain 3), GABARAP (GABA type A receptor associated protein), GABARAPL1 and GABARAPL2 (GABARAP-like)) to the lipid phosphatidylethanolamine in the growing phagophore membrane. The ATG8 proteins are thought to play an important role in cargo recognition, autophagosome closure and fusion with lysosomes.

In this review, we will focus on the first autophagy initiating complex: the ULK1 complex. What is this complex, how is it activated and how does it drive autophagosome formation? Though we do not have the full answers to all these questions, we will discuss our current understanding of how this protein complex is the key for autophagy initiation. For information on the other autophagy-related complexes, including the VPS34 complex and ATG8 conjugation machinery, the reader is encouraged to look at a recently published review [3].

## The core ULK1 complex: components, localization and function

ULK1 is a serine/threonine protein kinase and the mammalian orthologue of the yeast Atg1. There are five ULK1 homologues (ULK1, ULK2, ULK3, ULK4 and STK36 (serine/threonine kinase 36)); of these, only ULK1 and ULK2 are believed to be involved in conventional autophagy signalling. In most cell lines, loss of ULK1 is sufficient to disrupt autophagy; however, ULK2 is thought to act with a degree of redundancy in this pathway. This redundancy is highlighted by the need to knock out both ULK1 and ULK2 in mice to show the same neonatal lethality seen with loss of other core autophagy genes such as ATG5 and ATG7 [4–6]. ULK1 and ULK2 share approximately 52% protein sequence identity and 78% homology within their kinase domains, interact with the same core components and are believed to be regulated in a very similar manner. It is not clear why these two forms of ULK exist and it may be tissue expression levels that determine which kinase is dominant for autophagy induction. The importance of ULK1 in autophagy initiation has been highlighted in numerous studies. ULK1 was identified in HEK 293 cells where down-regulation of ULK1 was sufficient to inhibit autophagy [7], and mouse embryonic fibroblasts derived from ULK1/2 knockout mice are unable to induce autophagy in response to amino acid deprivation [6]. As might be expected for a protein kinase, ULK1’s kinase activity is essential for autophagy initiation: kinase-dead mutants of ULK1, as well as chemical inhibition of the ULK1 enzymatic activity, result in a block of autophagic flux [8–10].

In cells, ULK1 (and ULK2) appears to be constitutively in complex with at least three proteins: ATG13, FIP200 and ATG101 [11–15]. Interaction of ULK1 with ATG13 or FIP200 results in increased ULK1 kinase activity and stability [12–14]. Recently it has been shown that mice lacking FIP200 or ATG13 die *in utero*, and MEFs derived from these mice fail to initiate and complete autophagy, highlighting the importance of these proteins for autophagy progression [16–18]. This embryonic lethality is in contrast with mice lacking other core autophagy components, which as mentioned, die during the neonatal period. This suggests that ATG13 and FIP200 may have additional roles outside the ULK1 complex [16,17]. Indeed, ATG13 mutants that cannot bind to ULK1 or ULK2 were shown to partially rescue autophagy in ATG13-deficient cells [19]. Less is known about the third complex member, ATG101. ATG101 is also essential for autophagy and interacts with the ULK1 complex via direct binding to ATG13 [11,15].

The structure of the entire ULK1 complex has not yet been determined, though information on individual components is mounting. The crystal structure of the ULK1 kinase domain has been solved where it was found to adopt a relatively standard kinase fold, with the exception of a large loop between the N- and C-terminal lobes [20]. This study also identified an autophosphorylation site, at Thr^180^ in the activation loop, which had also been identified previously [21], and may be important for catalytic activity. Additionally, the crystal structure of ATG13 in complex with ATG101 was recently resolved—ATG13 is a heterodimer with ATG101 and bridges the interaction of ULK1 with FIP200 [22–24]. Biochemical data have also contributed to our understanding of complex architecture, with the ATG13-binding sites on ULK1 being mapped to ULK’s C-terminus [8], while ATG13 binds ULK1 also with its C-terminus [13,19]. Perturbation of these interacting sites leads to impaired autophagy, highlighting the importance of these protein–protein interactions.

The exact localization of the ULK1 complex under normal conditions is unclear, but upon amino acid starvation it forms punctate structures in close proximity to the ER (endoplasmic reticulum). These punctate structures co-localize with omegasomes, cradle-like structures of the ER that support autophagosome biogenesis [25]. The localization of ULK1 to these structures is one of the earliest observable events during autophagy initiation and occurs in conjunction with other factors such as the ER-localized transmembrane protein VMP1 as well as ATG9-decorated vesicles, the latter of which is discussed further below [26,27].

## Upstream regulation of the ULK1 complex

### Post-translational modifications

**Figure 2:**
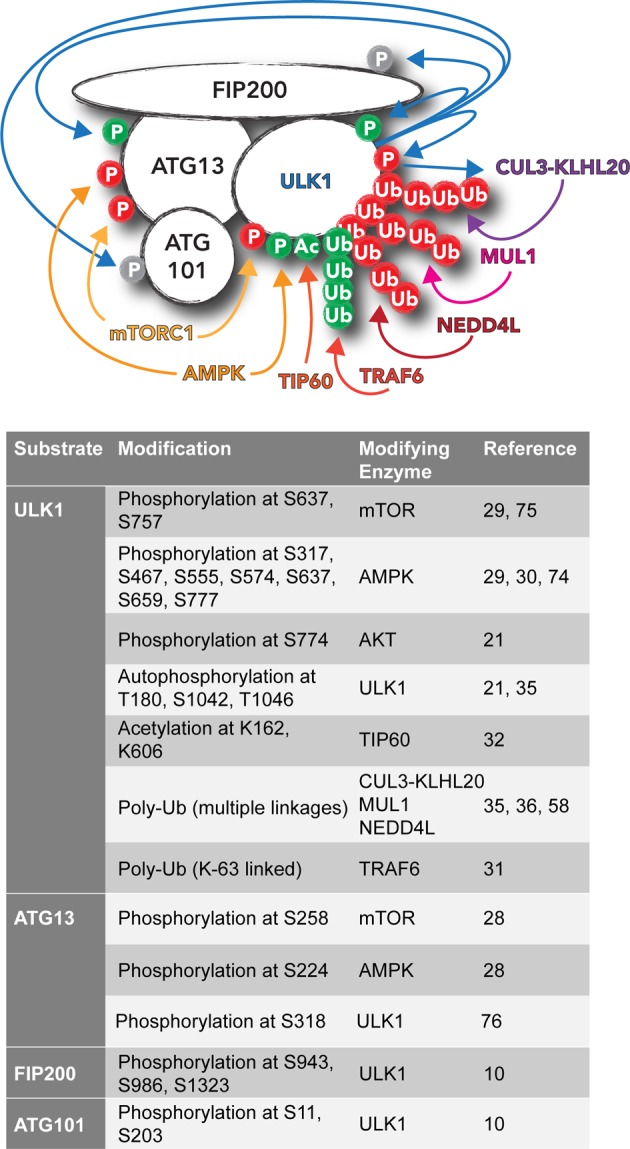
PTMs of the ULK1 complex The ULK1 complex undergoes multiple modifications: phosphorylation (P), acetylation (Ac) and ubiquitylation (Ub). Modifications coloured in green suggest activation, whereas in red inhibition, of the ULK1 complex and autophagy. The modifications and residues, as well as the responsible enzymes are listed below in the schema.

As can be seen from Figure 2, ULK1 undergoes an intricate and diverse set of post-translational modifications (PTMs), which emphasize the ULK1 complex’s role as a node to convert multiple stress signals into forming autophagosomes. Nutrient deprivation is a potent autophagy activator and the mechanism of amino acid starvation induced autophagy is by far the best characterized. Under these conditions, autophagy is rapidly initiated to drive bulk turnover of intracellular proteins and organelles, in order to provide a pool of amino acids to maintain synthesis of proteins essential for survival. A master regulator of autophagy in response to nutrient availability is the mTOR (mechanistic target of rapamycin) complex 1 (mTORC1), which is discussed in detail by Rabanal-Ruiz et al. in this issue [82]. mTOR is a serine/threonine protein kinase that is responsible for regulating cell growth and metabolism and becomes activated on the cytosolic side of lysosomes by a complex of proteins that sense amino acids. In the presence of amino acids, mTORC1 is active and inhibits autophagy by phosphorylating ULK1, as well as ATG13, at multiple residues [12–14]. Phosphorylation of ULK1 by mTORC1 results in suppression of its catalytic activity, thus inhibiting autophagy initiation. In a similar manner, mTORC1-dependent ATG13 phosphorylation also negatively influences ULK1 activity as well as complex translocation to autophagy initiation sites [28]. Upon amino acid deprivation, mTORC1 activation on the lysosomal surface is no longer maintained and both ULK1 and ATG13 are rapidly dephosphorylated, resulting in activation of the ULK1 kinase and concomitant autophagy induction.

mTOR activity is also fine-tuned by additional mechanisms, and as such, these modalities will also impinge upon ULK1 activation and autophagy. For example, growth factor signalling as well as energy deprivation can alter mTOR activity and hence autophagy. In case of the latter, low ATP levels or an increase in the AMP:ATP ratio, leads to activation of AMPK (AMP-activated protein kinase). AMPK can inactivate mTORC1 through phosphorylation of RAPTOR, a key protein present within the mTORC1. Importantly, AMPK can also directly phosphorylate and activate ULK1 at multiple serine residues [29,30]. In contrast, AMPK can phosphorylate ATG13, and in this instance acts synergistically with mTORC1 in an inhibitory fashion, in response to nutrient availability [28]. Therefore, the timing and context of these phosphorylation events are likely to be critical in determining the autophagic output.

mTORC1 has also been proposed to regulate ULK1 in a more indirect manner, by influencing its Lys^63^ (K63)-linked ubiquitylation and subsequent stabilization. mTOR can phosphorylate AMBRA1 (autophagy and beclin 1 regulator 1), a binding partner of the VPS34 complex, which inhibits its interaction with ULK1. Upon mTOR inhibition and autophagy induction, AMBRA1 brings the E3-ligase TRAF6 (TNF receptor associated factor 6) to ULK1 resulting in its K63-linked ubiquitylation, self-association and subsequent stabilization, thus enhancing ULK1 activity and autophagy induction [31].

In addition to mTOR, autophagy can also be regulated in response to growth factor removal by GSK3 (glycogen synthase kinase 3), which phosphorylates and activates the acetyltransferase TIP60 (60-kDa Tat-interactive protein). TIP60 can then directly acetylate ULK1, resulting in its activation [32]. Growth factors, namely insulin, can also regulate ULK1 through AKT-dependent phosphorylation, though the significance of this phosphorylation is currently unknown [21].

Given the high degree of ULK1 regulation by phosphorylation, it may come as no surprise that dephosphorylation of this complex is also critical. Though we do not yet have a complete picture of the phosphatases involved in regulating ULK1, two in particular have been implicated. Under starvation, protein phosphatase 2A (PP2A) in conjunction with its B55α regulatory subunit, has been shown to dephosphorylate ULK1 at Ser^637^ but not Ser^757^ (both mTOR-dependent sites) to promote autophagy [33]. Additionally, PPM1D (protein phosphatase 1D magnesium-dependent, δ isoform) has also been shown to dephosphorylate ULK1 at the same Ser^637^ in response to genotoxic stress, in order to promote ULK1 puncta formation and autophagy induction [34].

Protein ubiquitylation is another key PTM and, in addition to the K63-linked ubiquitylation mentioned above, ULK1 also undergoes modification by other ubiquitin linkages. These can target ULK1 for proteasomal degradation resulting in down-regulation of autophagy. ULK1 reportedly becomes ubiquitylated by the cullin E3 ligase complex, Cul3-KLHL20 (Cullin-3, kelch-like family member 20), and concomitantly degraded in a proteasome-dependent manner. During prolonged starvation, autophosphorylation of ULK1 at residues Ser^1042^ and Thr^1046^ results in recruitment of KLHL20, the substrate adaptor that allows Cullin-3-mediated ubiquitylation and concomitant degradation of ULK1, as well as ATG13 and components of the VPS34 complex. This terminates autophagy and prevents an excessive autophagic response [35]. Apart from Cul3-KLHL20, the E3-ligase NEDD4L (4-like E3 ubiquitin protein ligase) has also been suggested to regulate ULK1 degradation during prolonged starvation [36].

It is also important to note that in addition to protein degradation, ULK1 levels can be regulated by protein synthesis. At the transcriptional level, there are multiple factors that have been shown to regulate *ULK1* mRNA levels, which have been recently reviewed [37].

### Protein interactions

The interaction of ULK1 with its binding partners ATG13, FIP200 and ATG101 is essential for ULK1-dependent autophagy. However, it appears that ULK1 interaction network is much more elaborate and involves many different and diverse proteins. For example, a recent study suggests that IRGM (immunity-related GTPase family M protein), a small GTPase protein involved in the innate immune response, regulates AMPK activation and concomitantly AMPK-dependent ULK1 activation. Apart from this indirect role, it also binds to ULK1 and BECLIN-1 in order to mediate their assembly and promote autophagy in response to antimicrobial and inflammatory signalling [38]. Furthermore, C9orf72 (chromosome 9 ORF 72) a protein mutated in the neurodegenerative disorder ALS (amyotrophic lateral sclerosis), has recently been reported to regulate the ULK1 complex. In one study, C9orf72 was implicated in Rab1a (Ras-related protein Rab 1a)-dependent trafficking of ULK1 to autophagy initiating sites [39]. A different study suggests that C9orf72 regulates autophagy by affecting activity of mTOR, as loss of C9ofr72 results in inhibition of mTOR kinase activity and concomitant increase in autophagy [40]. Lastly, a third study suggests that C9orf72 is part of a multiprotein complex containing ATG101 and SMRC8 (Smith–Margenis syndrome chromosome region candidate 8), and functions in regulating expression and activity of ULK1 [41]. Here we should mention that SMCR8 has also been reported as a substrate of ULK1 in an independent study [42], although the exact role of C9orf72 and SMCR8 in the regulation of the ULK1 complex and autophagy remains to be determined. As is discussed below, ALS is not the only neurodegenerative disorder with a direct link to ULK1. Huntingtin, the protein mutated in Huntington’s disease, also binds to ULK1 where it is thought to act as a scaffold in regulating specific forms of autophagy [43].

## ULK1-dependent downstream regulation of autophagy

ULK1 kinase activity is essential for starvation-induced autophagy, implying the existence of key downstream substrates. Indeed, multiple proteins are now emerging as ULK1 substrates, though the exact significance of these to autophagy induction is still not clear in many cases (see Table 1). A phosphorylation consensus motif for ULK1 substrates has also been described, which should aid in further substrate identification [10].The first recognized ULK1 substrates were ULK1 complex members themselves, including ULK1 (autophosphorylation), ATG13, FIP200 and later on ATG101 [10,12–14]. As is shown in Figure 1, a clear downstream substrate of ULK1 is the VPS34 complex and the regulation of PI3P. A key study showing this, demonstrated that ULK1 directly phosphorylates BECLIN-1 at Ser^14^, leading to enhanced VPS34 activity, PI3P production and autophagy initiation [44]. Recently, ULK1 was shown to phosphorylate ATG14L at Ser^29^, another VPS34 complex member, again resulting in increased VPS34 activity and autophagy induction [45]. Finally, ULK1 has been shown to phosphorylate VPS34 itself at Ser^249^, though the exact function of this event is not clear [10]. The link between ULK1 and VPS34 does not stop here, as ULK1 also regulates the VPS34 complex through AMBRA1. AMBRA1 binds BECLIN-1 and mediates tethering of the VPS34 complex to the cytoskeleton. ULK1-mediated phosphorylation of AMBRA1, the sites of which have not yet been identified, results in its dissociation from the cytoskeleton and this is believed to regulate translocation of the VPS34 complex to autophagy initiation sites [46]. Taken together, these suggest a multistage regulation of the VPS34 complex by ULK1, potentially to enable a fine tuning or the robustness, of an autophagic response. In addition, the VPS34 complex is thought to act in a positive feedback loop with the ULK1 complex, with increased PI3P production leading to increased ULK1 recruitment, through a recently identified lipid-binding domain present in ATG13 in mammals [25].

**Table 1:** Published list of ULK1 substrates

Protein	Residue(s)	Function	References
**ULK1**	T180, S1042, T1046	T180 important for ULK1 kinase activity. S1042 and T1046 regulate ULK1 ubiquitylation and degradation during prolonged starvation	[21,35]
**ATG13**	S318 (S355 isoform 1)	S318 required for clearance of depolarized mitochondria	[74]
**FIP200**	S943, S986, S1323	Unknown	[10]
**ATG101**	S11, S203	Unknown	[10]
**ATG9**	S14	Promotes ATG9 trafficking in response to starvation	[50]
**BECLIN-1**	S14 (mouse)	Required for VPS34 activation	[44]
**AMBRA1**	S465, S635	Regulates dissociation of AMPRA1-VPS34-BECLIN-1 from the dynein complex, to promote interaction with ULK1 and autophagy	[10,46]
**ATG4B**	S316	Inhibits ATG4B activity and LC3 processing	[51]
**FUNDC1**	S17	Promotes mitophagy by enhancing FUNDC1 binding to LC3	[54]
**VPS34**	S249	Unknown	[10]
**ATG14L**	S29	Promotes autophagy by increasing VPS34 complex activity	[45]
**DENND3**	S554, S572	Activates Rab12 to facilitate autophagosome trafficking	[75]
**HK1**	S124	During amino acid and growth factor starvation in order to maintain homoeostasis of cellular energy and redox levels	[76]
**ENO1**	S282	During amino acid and growth factor starvation in order to maintain homoeostasis of cellular energy and redox levels	[76]
**PFK1**	S762	During amino acid and growth factor starvation in order to maintain homoeostasis of cellular energy and redox levels	[76]
**FBP1**	S63	During amino acid and growth factor starvation in order to maintain homoeostasis of cellular energy and redox levels	[76]
**Raptor**	S855, S859, S792	Inhibition of mTORC1 during starvation	[77]
**Sec23A**	S207, S312	Inhibition of ER to Golgi trafficking during starvation	[78]
**Cdc37**	S339	Disruption of Cdc37 client proteins stability	[79]
**Smrc8**	S400, S492, S562, T666	Unknown function in C9orf72 regulation of the ULK1 complex	[42]
**p62/SQSTM1**	S409	To promote aggregate clearance during proteotoxic stress	[43]
**Sting**	S366	To inhibit an excessive transcription of innate immune genes during their activation by cyclic dinucleotides	[80]

Abbreviations: DENND3, DENN domain containing 3; HK1, hexokinase 1; ENO1, enolase 1; PFK1, phosphofructokinase 1; FBP1, fructose-1,6-bisphosphatase 1; FUNDC1, FUN14 domain containing 1; p62/SQSTM1, sequestosome-1.

In addition to phosphorylation of the VPS34 complex, ULK1 regulates ATG9 trafficking during autophagy. ATG9 is currently the only transmembrane protein that has been found on the autophagosomal membrane and it also traffics the plasma membrane, *trans*-Golgi network and endosomes [47–49]. ATG9 is found to co-localize with forming autophagosomes in an ULK1-dependent manner, and it is thought to be involved in supplying membranes required for autophagosome biogenesis [49]. Recently, ULK1 has been found to act synergistically with the protein kinase SRC (SRC proto-oncogene, non-receptor tyrosine kinase), to phosphorylate ATG9, thus promoting translocation of ATG9-positive vesicles to the autophagy initiation sites [50].

ULK1 has also been implicated in regulating the ubiquitin-like conjugation machinery. A recently published study suggests that ULK1 mediates regulation of LC3 processing by phosphorylating the protease ATG4B, which is responsible for the conversion of pro-LC3 to LC3-I as well as converting LC3-II back into LC3-I[51]. Phosphorylation of ATG4B at Ser^316^ by ULK1 results in inhibition of ATG4B catalytic activity, though the precise consequences of this on autophagy induction are still not clear.

In addition to regulating autophagy initiation, it is possible that ULK1 also acts at later stages in the pathway, including autophagosome maturation. For example, a recent study in our laboratory reported that inhibition of ULK1 in cells, with the potent inhibitor MRT68921, resulted in a block in autophagy, but also the appearance of a small number of apparently ‘stalled’ autophagosomes, positive for early as well as late autophagosome markers [9]. The exact mechanism of this block remains to be determined, but this suggests that key, as yet unidentified, ULK1 substrates are present on these stalled structures and mediate autophagosome maturation.

## ULK1 and selective autophagy

Certain cargo can be specifically engulfed by the forming autophagosome in a process called selective autophagy. There are many types of selective autophagy reporter so far, including mitophagy (autophagy of mitochondria), aggrephagy (autophagy of protein aggregates) ER-phagy (autophagy of portions of the ER), pexophagy (autophagy of peroxisomes), xenophagy (autophagy of intracellular pathogens) and others [52]. Selective autophagy is discussed in detail by Lamark et al. [83] in this issue, and much of this area of autophagy has, quite rightly, been focused on how the specific cargoes are identified by the autophagy machinery. The role that the ULK1 complex plays here remains unclear, but nevertheless the autophagosome itself must be induced and ULK1 is likely to play a key role in this. One of the best studied pathways of selective autophagy is mitophagy, and under hypoxic conditions, ULK1 has been shown to translocate to the mitochondria. [30,53]. This mitochondrial recruitment of ULK1 is dependent on binding to the mitochondrial adaptor protein FUNDC1 (FUN14 domain containing 1). Once on the mitochondria, ULK1 phosphorylates FUNDC1 on Ser^17^, which is adjacent to the FUNDC1 LC3-interacting motif. This phosphorylation enhances FUNDC1–LC3 interaction to presumably aid engulfment of the mitochondrion [54]. Consistent with its role in energy sensing, AMPK and its phosphorylation of ULK1 has been implicated in ULK1 mitochondrial translocation [30,55].

One of the best characterized pathways for mitophagy is the PINK (PTEN-induced putative kinase 1)-Parkin pathway, which is impaired in some forms of hereditary Parkinson’s disease [56]. Briefly, the PINK-Parkin pathway is activated in response to mitochondrial depolarization and results in extensive ubiquitylation of numerous outer mitochondrial membrane proteins [57]. Ubiquitylated mitochondria are then recognized by autophagy adaptor proteins that target the engulfing autophagosome. Recently it was shown that that PINK1-Parkin-dependent mitophagy is mediated by the adaptor proteins NDP-52 (nuclear dot protein 52) and optineurin, which apart from being able to bind autophagosomes, were also shown to regulate ULK1 mitochondrial recruitment [81]. This suggests that ULK1 is recruited to directly initiate autophagosome formation at the mitochondrion, rather than triggering autophagosomes elsewhere and ‘capturing’ the ubiquitylated mitochondrion before autophagosome formation completes. It has also been reported that ULK1 partially translocates on to the mitochondria during selenite-induced mitophagy, where it becomes ubiquitylated by the mitochondrial E3-ligase MUL1 (mitochondrial E3 ubiquitin protein ligase 1). This modification results in proteasomal turnover of ULK1, but the role of this phenomenon during mitophagy is unclear [58].

As alluded above, Huntingtin can regulate selective autophagy by binding to ULK1 and the selective autophagy cargo receptor p62/SQSTM1 (sequestosome-1) [59]. Huntingtin had previously been linked to autophagy [60] but this recent study suggested that it can regulate different types of selective autophagy (mitophagy, lipophagy and aggrephagy), by binding to ULK1 and p62, and in doing so, dissociating ULK1 from mTOR and driving autophagosome formation on the selected cargo [59].

## Role of the ULK1 complex in human disease

Autophagy has a vital role in health maintenance. In general, autophagy is believed to be a cytoprotective mechanism that prevents the accumulation of damaged or impaired cellular components that could be detrimental to the cell if left to persist. Therefore, dysfunctional autophagy has been implicated in numerous diseases. There are many autophagy genes, mutations of which have strong correlation with human diseases. For example, a mutation in ATG16L1 has been linked to Crohn’s disease (CD), mutations in EPG5 (ectopic P-granules autophagy protein 5 homologue) have been associated with Vici syndrome and in many types of cancer BECLIN-1 is found monoallelically deleted [1]. So far, apart from a single nucleotide polymorphism (SNP) in ULK1 that is associated with CD [61], other disease-associated mutations of the ULK1 complex have not been reported, though altered ULK1 expression has been associated with cancer. ULK1 expression correlates with poor prognosis in nasopharyngeal [62], breast [63], colorectal [64] and gastric [65] cancers. The latter report showed that ULK1 down-regulation inhibited cell growth in these cell lines, suggesting that autophagy is utilized as a survival mechanism [65]. In a similar manner, knockdown of ULK1 during hypoxia results in cell death, implying that tumour cells, which are often present in hypoxic environments *in vivo*, rely on autophagy for their survival in an ULK1-dependent manner. The experimental evidence for involvement of ATG13, ATG101 and FIP200 in cancer is less clear, but given their critical role in regulating ULK1 function, they are likely to play a similar role.

mTOR inhibitors have been used in the clinic as an anticancer treatment, but efficacy has been surprisingly poor. As mTOR inhibition results in ULK1 and autophagy activation, this might give an advantage to cancer cells in order to survive drug treatment. Therefore, a combination therapy with mTOR inhibitors and autophagy inhibitors is a potential way to get around this perceived ‘drug resistance’. Indeed, various clinical trials are underway [1,66]. The recent identification of ULK1 inhibitors have expanded our potential arsenal in this endeavour [9,10]. Indeed, one of these studies showed that dual inhibition of ULK1 and mTOR promoted cancer cell apoptotic death [10].

## ULK1-independent autophagy

The ULK1 complex is essential for autophagy induction during amino acid starvation. However, it has been shown that MEFs lacking ULK1 and 2 are still able to induce autophagy during glucose starvation. In the present study, the authors show that ammonia production, due to amino acid catabolism, is responsible for ULK1-independent autophagy [6]. A later study showed that interaction between the ULK1 complex member FIP200 and ATG16L1 can determine ULK1-dependent or -independent autophagy. Here, the authors identified an interaction domain between FIP200 and ATG16L1, mutation of which abolished this interaction and amino acid induced autophagy, but not ammonia-induced autophagy [67]. Furthermore, it was recently suggested that the requirement for ULK1 in autophagy induction can be bypassed by an increase in VPS34 kinase activity, caused by inhibition of a newly identified and negative regulatory VPS34 acetylation event [68]. Further work is needed to clarify this, especially as ULK1 is likely to play additional roles outside VPS34 activation to orchestrate autophagosome formation. It is also worthy to note that ULK1 has been implicated in non-autophagy-related functions too [69].

## Conclusion

The ULK1 complex is an essential regulator of mammalian autophagy and the fact that its function is largely conserved throughout all eukaryotes, underlines its importance. However, we still do not fully understand the roles and regulation of this enigmatic complex, especially given the set of regulatory events described above. Many questions still remain, such as how the physical structure of the ULK1 complex allows it to perform its function? How or if, the ULK1 complex induces autophagy when mTOR is active? Perhaps some of the most important questions centre on when and where the identified pathways of ULK1 regulation are physiologically important. The vast majority of work in elucidating ULK1 function and mechanism has involved *in vitro* and *in cell* work; however, the rise of tractable animal models to study autophagy [70–73] will hopefully allow us to set the context of ULK1 regulation *in vivo*. These key tools will hopefully help us to determine the potential of ULK1, and autophagy in general, as a therapeutic target for the treatment of human pathologies.

## Summary

- The ULK1 complex consists of ULK1, FIP200, ATG13 and ATG101 and regulates initiation of autophagosome formation.
- Lack of one or more ULK1 complex components results in impaired autophagy in response to stimuli.
- ULK1’s serine/threonine protein kinase activity is required for starvation-induced autophagy.
- The mTORC1 pathway is a critical negative regulator of ULK1, and hence autophagy, in response to amino acid availability.
- The ULK1 complex can regulate selective autophagy and is involved in numerous human pathologies.
- ULK1 kinase activity is a potential target for the development of treatments targeting autophagy in disease therapy.

## Abbreviations

ALS: amyotrophic lateral sclerosis
AMBRA1: autophagy and beclin 1 regulator 1
AMPK: AMP-activated protein kinase
ATG: autophagy-related protein
ATG14L: ATG14-like
CD: Crohn’s disease
Cul3-KLHL20: Cullin-3, kelch-like family member 20
C9orf72: chromosome 9 ORF 72
ER: endoplasmic reticulum
FIP200: focal adhesion kinase family interacting protein of 200 kDa
FUNDC1: FUN14 domain containing 1
GABARAP: GABA type A receptor associated protein
K63: Lys^63^
LC3: microtubule-associated protein 1 light chain 3
MEF: mouse embryonic fibroblast
mTOR: mechanistic target of rapamycin
mTORC1: mTOR complex 1
p62/SQSTM1: sequestosome-1
PINK: PTEN-induced putative kinase 1
PI3P: phosphatidylinositol 3-phosphate
PTM: post-translational modification
TIP60: 60-kDa Tat-interactive protein
ULK1: unc-51-like kinase 1
VMP1: vacuole membrane protein 1
VPS: vacuolar protein sorting
